# Late Onset Anaphylaxis in a Hydatid Cyst Case Presenting with Chronic Urticaria

**DOI:** 10.1155/2013/658393

**Published:** 2013-07-11

**Authors:** Insu Yilmaz, Omur Aydin, Alexis Okoh, Zeynep Misirligil

**Affiliations:** ^1^Division of Immunology and Allergy, Department of Chest Diseases, School of Medicine, Ankara University, Dikimevi, 06590 Ankara, Turkey; ^2^School of Medicine, Ankara University, Ankara, Turkey

## Abstract

Hydatid cyst is still endemic in various regions of the world. It is the most frequent cause of liver cysts worldwide. Urticaria is sometimes the first manifestation of the disease. However anaphylactic reaction and urticaria have been very rarely reported in the literature. Traditionally, surgery has been the only accepted mode of treatment; however, percutaneous treatment has recently been proposed as an alternative. Cases of anaphylaxis have been reported after percutaneous drainage of hydatid cyst. However, anaphylaxis usually develops within a few hours. Herein, we describe the case of a patient who presented with hydatid cyst causing chronic urticaria and late anaphylactic reaction following percutaneous aspiration of a liver hydatid cyst. We emphasize that physicians should be aware of hydatid cyst as a possible etiology for seemingly chronic spontaneous urticaria, especially in endemic regions. Patients should be kept under observation for at least one day due to the risk of early and late anaphylaxis after percutaneous aspiration treatment.

## 1. Introduction

Urticaria is a heterogeneous group of diseases. All types and subtypes of urticaria share a common distinctive skin reaction pattern. Chronic urticaria (CU) is defined as urticaria that persists for more than six weeks and is further classified into subgroups such as physiologic, spontaneous, and other ones. Chronic persistent bacterial, viral, parasitic, or fungal infections have been suspected of triggering urticarial symptoms in patients with chronic spontaneous urticaria (CSU) [1]. One of these agents, the hydatid cyst (HC), is a parasitic infestation that is still endemic in most regions of the world and is still found in Turkey [2]. The HC is formed from the larvae form of the cestode *Echinococcus granulosus* and resides in the liver (65%) and lungs (25%). As anaphylactic reactions and allergic symptoms generally involve cases of HC rupture the treatment regimens for spontaneous cases have also been reported [3]. In this report, a case of CU with a liver HC and anaphylactic shock developing after percutaneous aspiration of the cyst is presented.

## 2. Case Report

**Figure 1:**
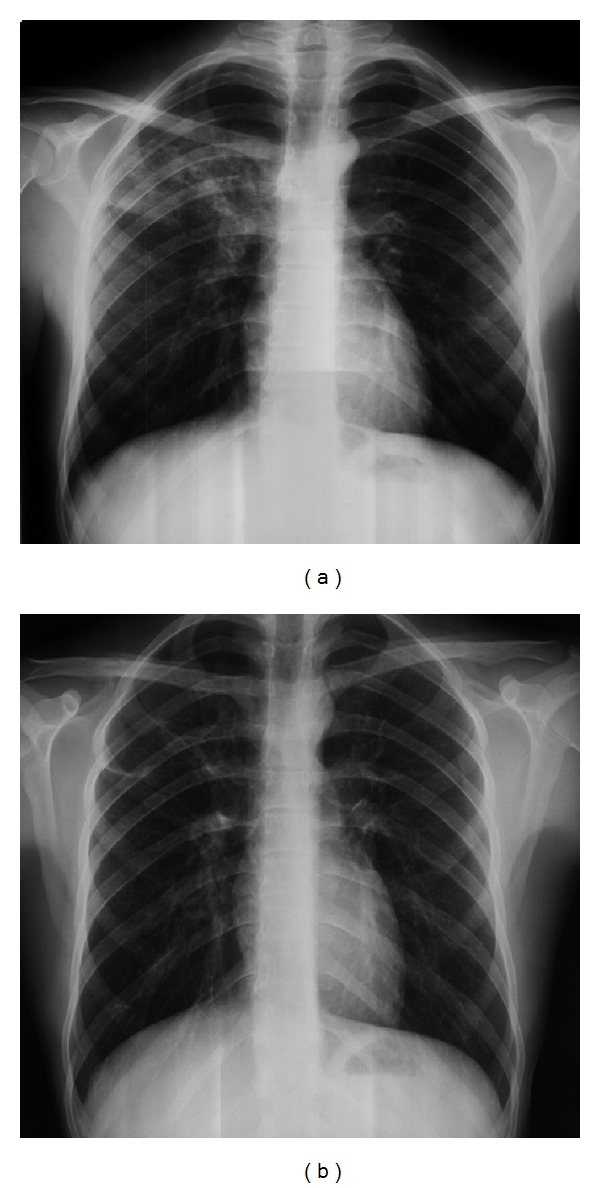
(a) Before antituberculosis treatment and (b) after antituberculosis treatment.

A 21-year-old male patient presented to our polyclinic with a 3-month history of urticaria. Urticaria treatment was started with pheniramine maleate of 22.7 mg at a twice daily dose in an outside clinic setting. A decrease in symptoms with this treatment was not seen and urticaria reoccurred on days when the patient did not take the antihistamines. The patient was therefore referred to our department for further investigations into the etiology of the urticaria. The patient, who appeared cachectic on presentation, had a complaint of a 4-5 kg weight loss in the last 6 months. Physical examination findings were blood pressure of 110/70, heart rate of 76/min, respiratory rate of 16/min, and body temperature of 37°C. Other physical examination findings were normal. Laboratory findings were as follows: leukocyte: 14800/mm^3^, sedimentation rate: 45 mm/hour, and C-Reactive Protein: 14.3 mg/dL. On chest X-ray a cavity with irregular borders and nonhomogenous infiltration was seen in the upper lobes (Figure 1(a)). A sputum acid fast test was positive. With the diagnosis of pulmonary tuberculosis, treatment with isoniazid (300 mg/day), rifampin (600 mg/day), pyrazinamide (1500 mg/day), and ethambutol (1500 mg/day) was started. Further tests conducted to identify the etiology of CU were specific IgE (with food and aeroallergens), thyroid autoantibodies, antinuclear antibody, vitamin B12, complete blood count, routine biochemistry, urinary analysis, stool analysis for parasites, and *Helicobacter pylori* antigens, and hepatitis markers were found to be normal. The patient was referred to a Tuberculosis Clinic. He was called for a control checkup six months after treatment and presented with a gain in weight and restoration of appetite but urticarial lesions continued in the patient whenever he did not take the antihistamine drugs. Chest X-ray showed a nearly resolved lesion (Figure 1(b)). An abdominal ultrasonography that was requested for investigation into the etiology of the urticaria showed a 55∗52 mm sized mature minimally fluid filled, capsuled HC with layered membranes in the posterior section of the right lobe of the liver. The patient was therefore referred to the Gastroenterology Department for further evaluation. On an abdominal computed tomography an approximately 6∗6 cm sized cystic lesion with thin internal septations and lobulizations was found in segment 7 of the anterior portion of the liver. With a positive HC indirect hemagglutination test (IHA) (1/256 titre) a diagnosis of HC was made and treatment with albendazole 400 mg/day was started. Percutaneous aspiration of the HC was performed after a week. The procedure was uneventful and the patient, who had normal vitals and no complications, was discharged an hour after observation in the hospital. After 7 hours the patient presented to the emergency service with generalized pruritis, urticaria, angioedema on the face, tongue and hands, dyspnea, cyanosis on the lips and dizziness. Treatment with adrenalin 0.5 mg (intramuscular), methylprednisolone 80 mg (intravenous), and pheniramine maleate 45.5 mg (intravenous) with intravenous hydration was started and the patient was hemodynamically stabilized. The patient presented to our clinic a day after the anaphylactic presentation. On examination his vital findings were normal. Physical findings included angioedema on the face around the eyes, erythematous and edematous uvula, and soft palate. A maintenance dose of methylprednisolone 40 mg/day, desloratadine 5 mg/day, and famotidine 40 mg/day was started and treatment continued for 5 days. At the end of the fifth day clinical presentations had completely resolved and systemic steroids and antihistaminic treatments were stopped.

## 3. Discussion 

Urticaria is a dermatologic reaction that can be caused by several different factors. Chronic urticaria is defined as urticaria that persists for more than six weeks [1]. In our patient as physiologic urticaria, other subtypes of urticaria such as cholinergic, aquagenic, contact, and exercise were excluded based on history. A diagnosis of CSU was made since a history of more than 6 weeks persisted. Food and drug related urticaria was excluded based on history and specific IgE tests. No pathologic findings were present in laboratory studies conducted to determine the etiology of CSU. A chest X-ray presentation compatible with tuberculosis and a positive acid fast test led to the diagnosis of tuberculosis and treatment was started. Chronic persistent bacterial, viral, parasitic, or fungal infections have been suspected of triggering urticarial symptoms in patients with CSU [1]. However, regression of urticaria was not seen after antituberculosis treatment even though the clinical and radiologic presentation of tuberculosis improved. As part of further investigations into the etiology of CSU an abdominal ultrasonagraphy was requested and showed a lesion in the liver compatible with HC. With a positive IHA, a diagnosis of HC was made. As anaphylactic reactions and allergic symptoms generally involve cases of HC rupture the treatment regimens for spontaneous cases have also been reported [3]. In our case urticaria lesions were present even though there was no evidence of rupture. Although speculative, this condition led us to believe that the antigenic components of the cyst, without cystic rupture, could have entered the systemic circulation through diffusion and cause a Type 1 sensitivity resulting in urticarial symptoms. This is because definitive tests targeted at *Echinococcus* specific IgE to support a HC mediated Type 1 hypersensitivity reaction causing urticaria in our patient were not conducted.

There are 3 different types of treatment for HC, medical treatment, surgical treatment, and percutaneous drainage. Traditionally, surgical treatment has been the most accepted method of treatment. However surgical treatment carries a high morbidity rate of 14%–60% and a mortality rate of about 7% [4]. The issue of medical treatment of an HC with only albendazole or mebendazole is controversial. Percutaneous drainage treatment has been used as an alternative treatment method to abdominal HC surgery for selected cases since the 1980s [5]. In recent years, instead of surgical removal of the cyst alone by surgical treatment, needle aspiration of the cyst and synchronous application of medical treatment have been suggested [6]. In our case, after a diagnosis of HC was made, treatment with albendazole was started and percutaneous drainage performed afterwards. Even though treatment with percutaneous drainage is commonly used most surgeons prefer open surgery due to fear of the risk of fluid leakage, dissemination, and anaphylaxis and this has caused open surgical treatment to take the place of percutaneous drainage [7]. However anaphylactic reaction is not the only complication of percutaneous drainage [7]. It can also be seen during surgical treatment [8]. Symptoms may present as mild urticaria or anaphylaxis [9]. In the current literature reported cases are generally of patients with early development of anaphylaxis [10, 11]. In our case percutaneous drainage was performed with no complications and anaphylaxis occurred seven hours after the procedure when the patient had been discharged from hospital. For this reason an allergist should be consulted for an allergy risk evaluation, before percutaneous drainage is performed in a patient who presents with a history of urticaria and is diagnosed with an HC. Furthermore these procedures should be performed in a well-equipped institution under intensive care conditions and patients should be kept under observation for at least a day before discharge. In our case the late presentation of HC anaphylaxis may have been due to late fluid leakage of the antigenic contents of the HC into the systemic circulation. 

## 4. Conclusion

Chronic spontaneous urticaria cases should be carefully investigated for chronic bacterial, viral, fungal, and parasitic infections. Particularly in regions where HC is endemic, HC as an etiology should be kept in mind. Due to the high risk of anaphylaxis that can develop during procedures in the treatment of HC, procedures of this sort should be conducted in settings that may possibly require emergency interventions with adrenaline and have intubation sets available. Patients should be kept under observation in a clinical setting for at least a day before being discharged due to the possibility of the development of late anaphylactic reactions.
